# Does Spiritual Group Psychotherapy Impact on The Rate of Pregnancy? A Case Report

**Published:** 2012

**Authors:** Leili Mosalanejad, Anahita Khodabakshi Koolaee, Fahime Shoyokh

**Affiliations:** 1 Department of Mental Health Education, Jahrom University of Medical Sciences, Jahrom, Iran.; 2 Department of Family Counseling, University of Social Welfare and Rehabilitation Science, Tehran, Iran.

**Keywords:** Infertility, Pregnancy, Spiritual psychotherapy

## Abstract

Distressing character of infertility has led to the development of several psychosocial interventions. We describe two women who suffered from infertility with unknown cause for a long-time period. They participated in weekly, one and half hour group psychotherapy for 10 sessions regularly and became pregnant after these sessions. Group psychotherapy can be used for infertile women who suffer from infertility with unknown cause.

## Introduction

Infertility is a complex crisis of life that is a psychological threat (1,2). The association of stress and infertility in humans is still unclear (3,4). Psychological treatment techniques are known to not only prevent and lessen various mental problems but also to play a positive role in physical health and a successful pregnancy (5-8).

Medications applied for psychiatric disorders may increase the likelihood of somnambulism in adults (4).Sleepwalking is mostly seen when alcohol is consumed with anti-psychotic and sedative medications, anti-depressants, antihistamines and stimulants (2).Psychiatric disorders such as depression, bipolar disorder and schizophrenia are also associated with parasomnias and they may increase the chance of sleepwalking (4).The following article is a case report of The Association for Spirituality and Psychotherapy (ASP) aims to advance the field of psycho-spiritual therapy by establishing a wide range of psychological theories and spiritual traditions (9). It is applicable in the cases where people have to deal with stress-provoking situation causing a crisis of meaning in their lives (10).

Spiritual group psychotherapy is to listen to the material provided in a group session with an ear for themes having to do with greater meaning, feelings of transcendence and high aspirations.

Data gathering is done using two questionnaires. The Depression, Anxiety and Stress Scale (DASS-21) are a 21-item instrument designed to measure three negative affective states of depression, anxiety, and stress (11,12). The Penn State Worry Questionnaire (PSWQ) investigates clinical and non-clinical groups of adults. This tool consisted of 16 Likert items (13). These two questionnaires have been reported to have excellent reliability and validity and have been normalized in Iranian society (12, 13).

The aim of this case report is to show the unique impact of spiritual psychotherapy on pregnancy.

## Case Report

The first case was a 39-year-old woman whose marriage date was 11 years ago. She suffered from infertility with unknown cause. Diagnostic testswere normal. She presented concerning about her age and fear about not having any child or having a child with mental retardation due to advanced age pregnancy.

Before enrolment, pre-test using the DASS-21 and PSWQ questionnaires were done. She participated regularly in group psychotherapy sessions and presented her feelings about her problems. Table 1 presents the objectives of the performed sessions. After 12 sessions, she did not present any more. Upon contact with her it appeared that she had become pregnant and her obstetrician advised her to have complete bed rest. The post-test was done at her home. Total DASS-21 score on pre-test was 46 (depression = 14, anxiety=17, stress= 15). Total DASS-21 score on pre-test decreased to 27 (depression = 8, anxiety = 9, stress = 10,). The PSWQ score also decreased from 22 on pre-test to 11 on post-test. On the last follow-up, she was pregnant at 16 weeks of gestation and according to amniocentesis performed the fetus was healthy.

The second patient was a 25-year-old woman whose marriage date was 8 years ago. She suffered from infertility with unknown cause. Hormonal and diagnostic tests were normal. Due to sever generalized anxiety disorder and major depression disorder observed by her obstetrician, she was referred to psychologist. She had severed depression with depressed appearance, irritability, continuous crying, helplessness, insomnia and loss of communication. She became candidate to participate at group psychotherapy sessions. Due to sever anxiety and depression observed by psychologist (according to the DSM IV-TR criteria), in the first session she received personal counseling. Then, she participated at group psychotherapy sessions continuously. Total score of the DASS-21 scale was 41 (depression= 12, anxiety= 15, stress = 14) and when finished the psychiatric approach the total score decreased to 30 (depression = 8, anxiety = 12, stress = 10). The PSWQ score also decreased from 18 on pre-test to 11 on post-test. Five months after completing group psychotherapy sessions she became pregnant.

## Discussion

Resent research showed that spiritual group psychotherapy for infertile women can increase the rate of pregnancy. However, review of the literature revealed controversies on psychological treatment in infertile participants (14)

.Strauss et al. conducted a meta-analysis of the comparative efficacy of psychological interventions in group settings and reported that higher conception rates among patients following psychotherapeutic intervention (15). Others reported that psychiatric intervention uncover psychological conflicts in infertile women and affect the rate of pregnancy (16).

Emery et al. reported that after infertility counseling, intensity of child wish decreased and pregnancy rate increased (17). Conversely, many studies have reported the efficacy of psychological interventions on infertile women to cope with stress (18).

On the basis of the results, psychological interventions are beneficial for infertile patients, but more randomized controlled trials are needed.

**Table 1:** Objectives of group psychotherapy sessions

- Identifying goals and rules of meeting.
- Recalling their capabilities that other people have forgotten.
- Identifying anxiety-related factors and all strategies to expose to them.
- Identifying assisted reproductive therapy and providing information about the types of treatment.
- Necessary to maintain one's personal identity to find the meaning of love.
- How to establish good relations with families.
- Finding the hidden meaning of the infertility problem through emphasizing the philosophy of life and marriage.
- Recognizing the value of creativity.
- Creating the kind of work which can give life meaning (helping charities, e.g. organ donation).
- Understanding the empirical values the meaning of life has and its value in addressing the nature.
Trend values:
Talk about situations where people have no power to deal with them.

## Authors’ Contributions

LM involved to apply the psychological approach in group, the reviewing the scientific literature, acquisition of clinical data, conceived and designed the evaluation, interpreted them and helped to draft the manuscript. AKK participated in revision of the manuscript. All authors read and approved the final manuscript. Group psychotherapy was performed by a LM weekly in one and half hour duration 10 sessions.

## Notes

**Declaration of interest:** None.
